# Steep Switching of In_0.18_Al_0.82_N/AlN/GaN MIS-HEMT (Metal Insulator Semiconductor High Electron Mobility Transistors) on Si for Sensor Applications †

**DOI:** 10.3390/s18092795

**Published:** 2018-08-24

**Authors:** Pin-Guang Chen, Kuan-Ting Chen, Ming Tang, Zheng-Ying Wang, Yu-Chen Chou, Min-Hung Lee

**Affiliations:** 1 Institute of Electro-Optical Science and Technology, National Taiwan Normal University, Taipei 11677, Taiwan; 2 National Nano Device Laboratories, Hsinchu 30078, Taiwan; 3 Device Design Division, PTEK Technology Co., Ltd., Hsinchu 30059, Taiwan

**Keywords:** InAlN, swing, wafer-scale, high-electron-mobility transistor (HEMT)

## Abstract

InAlN/Al/GaN high electron mobility transistors (HEMTs) directly on Si with dynamic threshold voltage for steep subthreshold slope (<60 mV/dec) are demonstrated in this study, and attributed to displacement charge transition effects. The material analysis with High-Resolution X-ray Diffraction (HR-XRD) and the relaxation by reciprocal space mapping (RSM) are performed to confirm indium barrier composition and epitaxy quality. The proposed InAlN barrier HEMTs exhibits high ON/OFF ratio with seven magnitudes and a steep threshold swing (SS) is also obtained with SS = 99 mV/dec for forward sweep and SS = 28 mV/dec for reverse sweep. For GaN-based HEMT directly on Si, this study displays outstanding performance with high ON/OFF ratio and SS < 60 mV/dec behaviors.

## 1. Introduction

Wide-bandgap GaN-based HEMTs have attracted lots of attention due to sensor applications for gas, pH, and biomedical analyses, etc. [1,2]. How to lower the operation voltage by using steep switching technology is a critical issue in the Internet of Things (IoT) era, which is beneficial for reducing power consumption and improving reliability. Recently, GaN HEMTs based directly on Si have shown advantages and benefits for larger area wafer-scale epitaxy for high throughput mass-production. The incorporation of indium into GaN as a barrier layer improves the power density and reliability because of lattice match for mole fraction ~18% [3,4], as well as higher polarization than possible with the general AlGaN barrier [5]. The strong spontaneous polarization of InAlN/GaN leads higher 2DEG charge density and drive-current as compared with general AlGaN/GaN. A lattice-matched InAlN/GaN configuration possessing high chemical and thermal stability is reported with high-temperature 1-MHz large-signal operation at 1000 °C (in vacuum) for 25 h [6]. The InAlN HEMTs thus offer the opportunity of use in environments with temperatures of at least 1000 °C. This characteristic makes high temperature sensor applications feasible InAlN barrier metal insulator semiconductor high electron mobility transistors (MIS-HEMTs) on sapphire have been demonstrated by the Hong Kong University of Science and Technology (HKUST) [7] with Schottky source/drain with steep subthreshold swing (SS) behavior and high ON/OFF ratio. The InAlN/GaN on Si with ~10^7^ and 10^8^ ON/OFF ratio with Ohmic and hybrid source/drain, respectively, is reported [8,9]. Intel exhibits a near ideal 60 mV/dec of subthreshold swing for MIS-HEMT enhancement mode, and a depletion mode device with steep SS < 60 mV/dec because of “negative” capacitance effect is shown using an AlInN metal-oxide-semiconductor (MOS) HEMT on SiC [10]. The negative capacitance concept is already demonstrated for steep switching on the Complementary Metal-Oxide-Semiconductor (CMOS) platform, including experimental and simulation development [11]. In general, the barrier layer with incorporated In exhibits a steep switch slope, ultra-low drain current leakage floor, and high ON/OFF ratio when compared with AlGaN barriers.

In this study, the In_0.18_Al_0.82_N/AlN/GaN directly on Si substrate with dynamic threshold voltage effect for steep switch slope characteristic is demonstrated. The advantages of InAlN HEMTs grown directly on a Si substrate are not only high thermal dissipation, but also high throughput, CMOS-compatible wafer-scale, and low cost, as compared to SiC or sapphire. It should be noted that the thermal conductivity of Si (~1.3 W/cm °C) and SiC (~3.6 W/cm °C) are much higher than sapphire (~0.23 W/cm °C).

## 2. Device Fabrication

**Figure 1:**
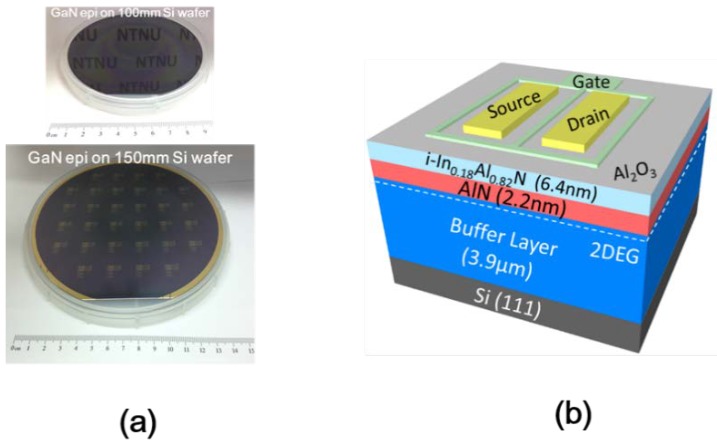
(**a**) GaN-based grown directly on 100 mm and 150 mm Si(111) wafer for CMOS compatible wafer-scale standard. (**b**) Schematic diagram of InAlN/AlN/GaN-on-Si MOS-HEMT.

**Figure 2:**
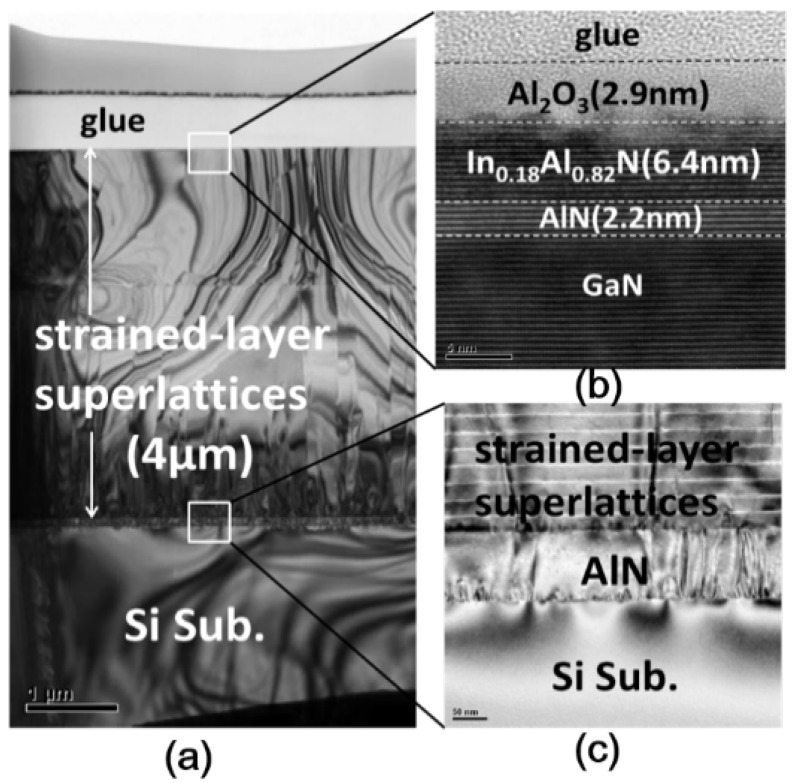
(**a**) GaN-based grown directly on 100 mm and 150 mm Si(111) wafer for CMOS compatible wafer-scale standard. (**b**) Cross-sectional TEM of 2.9 nm-thick Al_2_O_3_ cap layer and In_0.18_Al_0.82_N(6.4 nm)/AlN(2.2 nm) barrier layer. (**c**) Strained-layer superlattices/AlN on Si substrate.

The InAlN/AlN/GaN HEMTs structure is grown on the 150 mm/100 mm Si(111) substrate by Metal Organic Chemical Vapor Deposition (MOCVD, Figure 1a), and the schematic diagram of InAlN/AlN/GaN-on-Si MOS-HEMT as shown in Figure 1b. An about 3.9 μm-thick carbon-doped buffer layer and 300 nm i-GaN are deposited. Subsequently, a 2.2 nm AlN spacer is formed to reduce alloy scattering and interface roughness [12]. A strained-layer superlattices (SLS) structure TEM image shown in Figure 2a,c shows several secondary peaks by SLS. Note that the SLS is composed of AlN and GaN supercycles. The purpose is strain relaxation and dislocation pinning at the SLS buffer layer to obtain perfect InAlN/GaN epitaxy. The 6.4 nm barrier layer is grown with In_0.18_Al_0.82_N on the top to form two-dimensional electron gas (2DEGs), as shown in Figure 2b. A capping layer of approximately 2.9 nm Al_2_O_3_ is formed as a gate dielectric and prevents the barrier layer oxidation during the source/drain rapid annealing process. Moreover, the Al_2_O_3_ passivation can improve current collapse at saturation region [13], which is performed for 30 cycles by atomic layer deposition (ALD) using a Fiji-202 DCS (Cambridge NanoTech, Waltham, MA, USA) at 250 °C with trimethyl-aluminium (TMA) and H_2_O as the precursors. For the fabrication process of the devices, the gate-last process is performed. The Ohmic source/drain contacts are placed by the liftoff technique, in which Al_2_O_3_ cap layer is removed in the same layout of lithography step. Ti/Al/Ni/Au (20 nm/120 nm/25 nm/100 nm) is then deposited by Electron-Beam Evaporator with working pressure <4.0 × 10^−6^ Torr. After liftoff processing and cleaning the residual photoresist, rapid thermal annealing (RTA) at 850 °C for 30 s in high purity N_2_ ambient is performed to form Ohmic contact.

## 3. Results and Discussion

**Figure 3:**
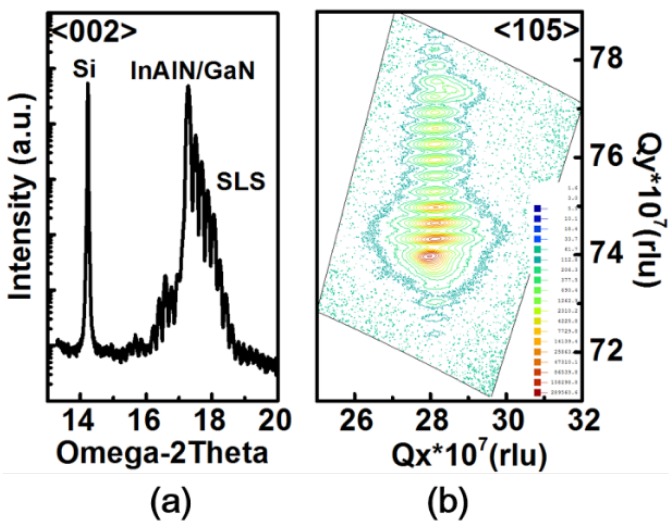
(**a**) High-Resolution X-ray Diffraction (HR-XRD) rocking curve of the (002) peak of InAlN/AlN/GaN-on-Si. The signal peaks of the Si substrate and InAlN or GaN are observed, indicating the Indium-based ternary heterojunction structure grown directly on the Si substrate. (**b**) RSM (Reciprocal Space Mapping) of InAlN/AlN/GaN on-Si in (002). The peaks are aligned, indicating full relaxation in GaN with a SLS buffer layer and strain free In_0.18_Al_0.82_N/GaN.

**Figure 4:**
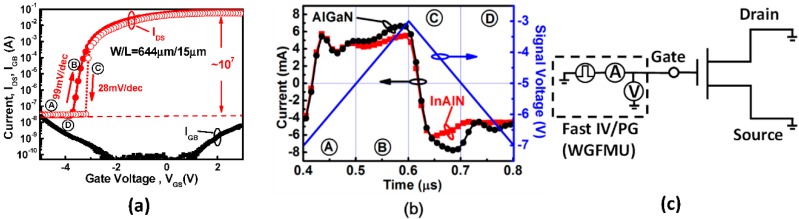
(**a**) The transfer characteristic (*I_DS_V_GS_*) of AlGaN/GaN-on-Si and InAlN/AlN/GaN-on-Si MOS-HEMTs. The InAlN device has I_ON_/I_OFF_ ~10^7^ and SS = 28 mV/dec covering up to ~4 decades in the reverse sweep. The *I_GB_* is lower than *I_DS_*. (**b**) Transient current of an AlGaN and InAlN device. The asymmetric current with signal up and down is due to intrinsic spontaneous polarization. (**c**) The measurement setup of transient response by using the waveform generator/fast measurement unit (WGFMU) module.

**Figure 5:**
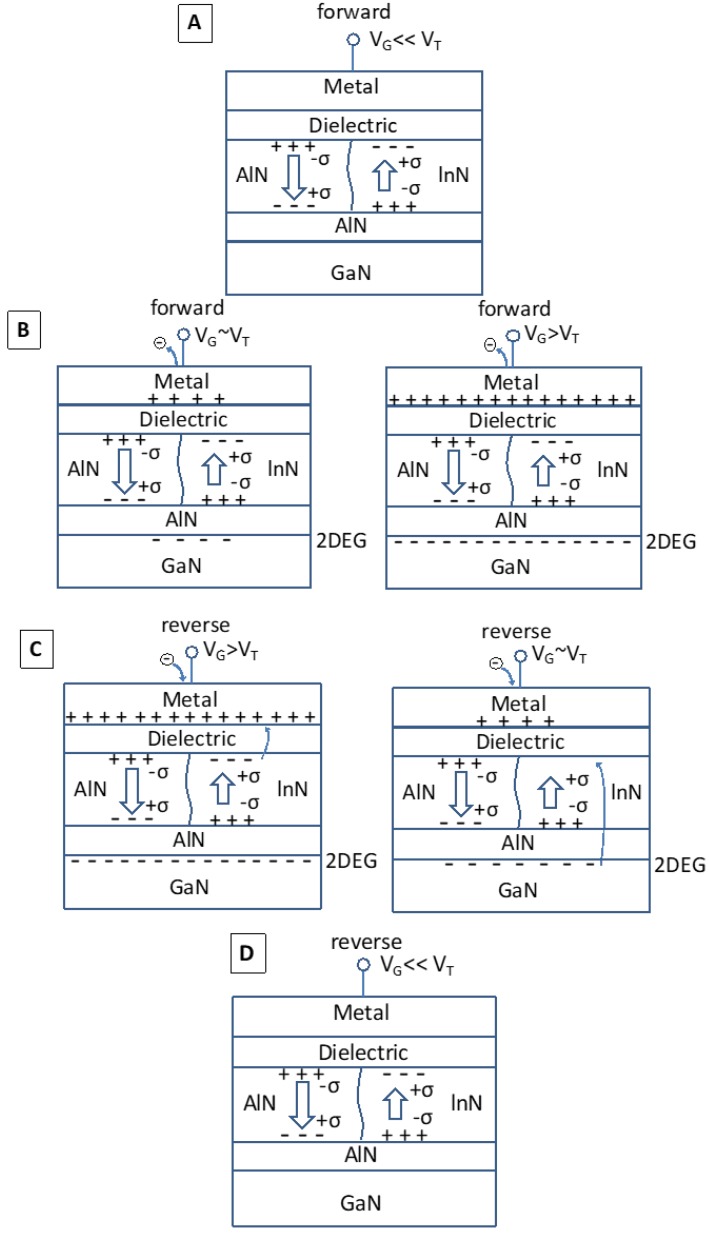
Schematic diagram showing the charge balance at case (**A**–**D**) in Figure 4. For case C, electrons of acceptor-like traps (Q_A_) transit to metal electrode and lead to drive out the electrons of 2DEG for reverse sweep. This results in *V_T_* being more dynamically positive and SS below 60 mV/dec for reverse sweep.

The sheet resistance and electron mobility of 2DEG obtained in In_0.18_Al_0.82_N/AlN/i-GaN are 527.7 ohm/□ and 820 cm^2^/Vs, respectively, by Hall measurement. The composition of indium = 18% is confirmed from the (002) reflection by HR-XRD as shown Figure 3a, and the same position for InAlN and GaN indicates the lattice-match. Note that the Si peak is the reference and represents InAlN/GaN growing directly on the Si substrate. The multiple peaks indicate a strained-layer superlattice as graded buffer layer. RSM in the (002) and (105) reflection direction indicates strain-free In_0.18_Al_0.82_N/GaN and full relaxation in GaN with a graded buffer layer, respectively, as shown in Figure 3b. The electrical characteristics are performed by a Keithley 4200 semiconductor parameter analyzer—with high power source measure units (SMUs). The transfer characteristics (*I_DS_V_GS_*) are shown in Figure 4a with high ON/OFF ratio ~10^7^ for InAlN device. The off-state current is ~2 × 10^−8^ A/mm (i.e., 2 × 10^−11^ A/μm) with a low leakage current because of the lattice-match between In_0.18_Al_0.82_N and GaN, which is close to the limitation of the measurement instrument and environment (~10^−12^–10^−15^ A/μm). Based on Vegard’s Law, the lattice is matched and strain free between the In_0.18_Al_0.82_N and GaN heterojunction [14]. The *I_GB_* is ~10^−8^–10^−12^ A, which is lower than transient current in Figure 4b. Therefore, the gate transient current corresponding to triangular voltage stimulus is contributed by displacement current. The low *I_GB_* is due to Al_2_O_3_ as gate dielectric for MOS-HEMT. The saturation drain current (*I_Dsat_*) of InAlN device is measured ~125 mA/mm with *L_G_* = 15 μm at *V_DS_* = 10 V and *V_G_* = 2 V. A steep SS that is also obtained in the InAlN device exhibits SS = 99 mV/dec for forward sweep and SS = 28 mV/dec for reverse sweep. For gate bias smaller than *V_T_*, the channel is not formatted due to no 2DEG. The electrons accumulate on top of GaN to form 2DEG for the channel with gate bias approaching to *V_T_*, as shown in Figure 5. The fast-current response for transient behaviors between the gate and source/drain shows similarly for InAlN and AlGaN in Figure 4b. The measurement setup is shown in Figure 4c, and the waveform generator/fast measurement unit (WGFMU) module is used. The triangular waveform is applied as blue line in Figure 4b. The voltage range is from −7 V to −3 V to correspond OFF-state to subthreshold region of *I_DS_V_GS_* in Figure 4a. The gate response current is shown in black and red line in Figure 4b for displacement current, in which it is much higher than DC gate leakage (Figure 4a). Note that the transient current response to the triangular voltage stimulus is used for ferroelectric material polarization by displacement current extraction [15].

With increasing bias, a lower displacement current in InAlN is observed for cases B due to neutralized spontaneous polarization of AlN (Al-rich) and InN (In-rich). For reverse sweep with case C, electrons of acceptor-like traps (Q_A_) transit to metal electrode as shown in Figure 5 and lead lower displacement current in Figure 4b. This would make the electrons transit from 2DEG to Q_A_ at Al_2_O_3_/InAlN interface of InN region for reverse sweep to have gate bias approach to V_T_. This results in *V_T_* being more dynamically positive and SS below 60 mV/dec for reverse sweep in Figure 4a. Finally, the 2DEG is vanished with a gate bias smaller than *V_T_* and back to the initial state. Note that the higher transient current of AlGaN in case D reflects the higher leakage current in Figure 4b. The asymmetric current with signal up and down (cases A & B vs. C & D in Figure 4b) is because of intrinsic spontaneous polarization in barrier layers. The steep switching in this work is obtained by displacement charge transition effect, which is different with other steep slope transistors technology, such as negative capacitance, threshold selector, TFET (tunneling FET), etc. For negative capacitance, the surface potential or internal gate voltage is amplified by ferroelectric gate stack [16]. The spontaneous rupture of filament is developed in Ag/TiO_2_-based device for threshold selector [17]. For TFET, the steep current increasing is occurred by BTBT (band-to-band tunneling) [18].

**Figure 6:**
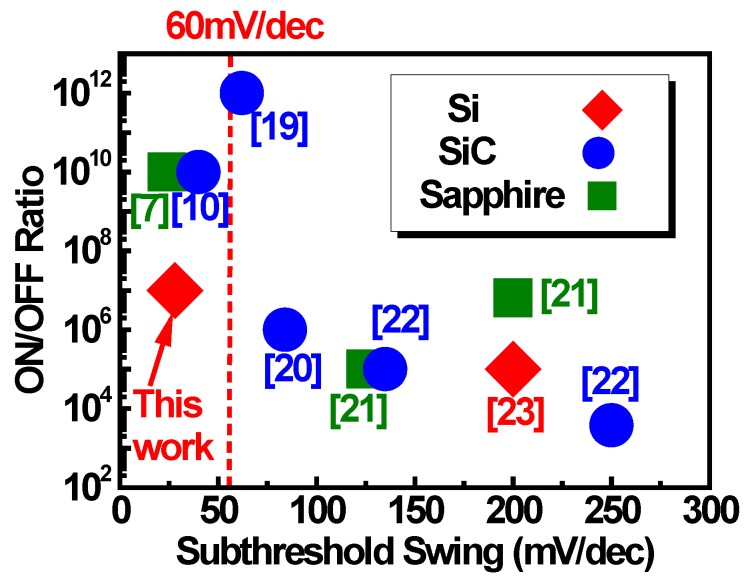
ON/OFF ratio vs. subthreshold swing of GaN-based devices on Si, SiC, and Sapphire substrates. This study shows SS <60 mV/dec of InAlN barrier GaN MOS-HEMT first time directly-on-Si.

Figure 6 summarizes the GaN-based devices on Si, SiC, and sapphire substrates for ON/OFF ratio and subthreshold swing [7,10,19,20,21,22,23]. This study demonstrates the InAlN barrier GaN MOS-HEMT for SS <60 mV/dec (reverse sweep SS = 28 mV/dec) with the first time directly-on-Si and outstanding performance with high ON/OFF ratio (~10^7^). Besides, the ON/OFF ratio of InAlN barrier GaN MOS-HEMT directly-on-Si can be further improved with Schottky-drain contact technology to 10^8^ [9]. Comparison with different substrate and structure are shown in Figure 6. A steep SS, ultra-low I_OFF_, and high ON/OFF ratio of InAlN/GaN on-Si MIS-HEMT are achieved.

## 4. Conclusions

The heterojunction of In_0.18_Al_0.82_N and GaN with lattice-match is validated by HR-XRD and RSM to confirm the indium barrier composition and epitaxy quality. The proposed promising wafer scale InAlN/Al/GaN HEMT directly-on-Si with steep subthreshold slope (SS < 60 mV/dec) is demonstrated in this study and is attributed to dynamic threshold voltage effect. The performance of the InAlN barrier HEMTs exhibits high ON/OFF ratio with seven magnitudes, and a steep SS is also obtained with SS = 99 mV/dec for forward sweep and SS = 28 mV/dec for reverse sweep. For the on-Si device, this study displays outstanding performance with high ON/OFF ratio and SS < 60 mV/dec behaviors. The steep slope characteristics of InAlN HEMTs growth on a Si substrate is feasible for applications, such as gas, pH, biomedical sensors, etc., and it is beneficial for reducing power consumption and reliability improvement in the IoT era.
